# Serum metabolomic analysis reveals key metabolites in drug treatment of central precocious puberty in female children

**DOI:** 10.3389/fnmol.2022.972297

**Published:** 2023-01-27

**Authors:** Guo-you Chen, Li-zhe Wang, Yue Cui, Jin-cheng Liu, Li-qiu Wang, Long-long Wang, Jing-yue Sun, Chang Liu, Hai-ling Tan, Qi Li, Yi-si Jin, Zhi-chun Xu, De-jun Yu

**Affiliations:** ^1^The Fifth Affiliated Hospital of Harbin Medical University, Women and Children’s Healthcare Hospital, Daqing, China; ^2^College of Pharmacy, Daqing Campus, Harbin Medical University, Daqing, China; ^3^Heilongjiang Provincial Hospital, Harbin, China

**Keywords:** central precocious puberty, drug treatment, key metabolites, metabolomics, serum of female children

## Abstract

Precocious puberty (PP) is a common condition among children. According to the pathogenesis and clinical manifestations, PP can be divided into central precocious puberty (CPP, gonadotropin dependent), peripheral precocious puberty (PPP, gonadotropin independent), and incomplete precocious puberty (IPP). Identification of the variations in key metabolites involved in CPP and their underlying biological mechanisms has increased the understanding of the pathological processes of this condition. However, little is known about the role of metabolite variations in the drug treatment of CPP. Moreover, it remains unclear whether the understanding of the crucial metabolites and pathways can help predict disease progression after pharmacological therapy of CPP. In this study, systematic metabolomic analysis was used to examine three groups, namely, healthy control (group N, 30 healthy female children), CPP (group S, 31 female children with CPP), and treatment (group R, 29 female children) groups. A total of 14 pathways (the top two pathways were aminoacyl–tRNA biosynthesis and phenylalanine, tyrosine, and tryptophan biosynthesis) were significantly enriched in children with CPP. In addition, two short peptides (His-Arg-Lys-Glu and Lys-Met-His) were found to play a significant role in CPP. Various metabolites associated with different pathways including amino acids, PE [19:1(9Z)0:0], tumonoic acid I, palmitic amide, and linoleic acid–biotin were investigated in the serum of children in all groups. A total of 45 metabolites were found to interact with a chemical drug [a gonadotropin-releasing hormone (GnRH) analog] and a traditional Chinese medicinal formula (DBYW). This study helps to understand metabolic variations in CPP after drug therapy, and further investigation may help develop individualized treatment approaches for CPP in clinical practice.

## Graphical Abstract

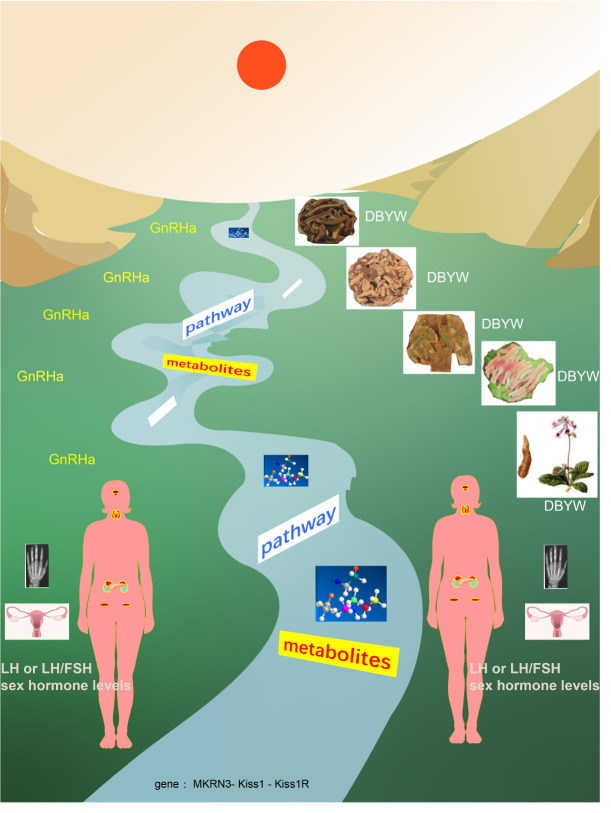

## 1. Introduction

In recent years, the incidence of precocious puberty (PP) has increased among children, primarily girls, in several Western European countries (Parent et al., 2003), which has drawn considerable attention to PP in other countries as well (Aksglaede et al., 2009; Su et al., 2020). In China, PP is frequently encountered by pediatricians, a large number of families, and healthcare personnel. Therefore, PP has become an intractable problem (Bessa et al., 2017a; Guo et al., 2021; Liu et al., 2021). Several central and peripheral mechanisms underlie the pathogenesis of PP (Klein et al., 2017). One of the causes of PP is the early activation of pulsatile gonadotropin-releasing hormone (GnRH) secretion (central precocious puberty, CPP) owing to hypothalamic tumors, lesions, or genetic conditions; however, the specific etiology remains unclear (Su et al., 2020). Approximately 10–20% of girls and a majority of boys have underlying pathological causes, whereas some may have idiopathic etiology (idiopathic PP) (Srensen et al., 2012). PP can cause distress to the patients, guardians, and caregivers of children (Wei et al., 2017). During childhood, PP is easily overlooked and provokes self-consciousness, therefore making the patients vulnerable to psychological disorders, such as low self-esteem and loneliness (Kim and Park, 2009), and the complications of PP continue to affect patients even after they grow old. Therefore, it is necessary to identify critical targets of PP and understand the mechanisms underlying its pathogenesis in childhood. Recent studies have identified some possible gene markers for PP. Sirtuin 1 (SIRT1)-mediated inhibition of Kiss1 is a key epigenetic mechanism associated with nutrition and obesity in mammalian puberty (Vazquez et al., 2018). A high frequency of MKRN3 mutations is observed in boys with CPP (Bessa et al., 2017b). Prepubertal exposure to dietary relevant levels of zearalenone (ZEA) can induce CPP in female rats *via* premature activation of the hypothalamic kisspeptin–GPR54–GnRH signaling pathway (Yang et al., 2016). However, the key molecular mechanisms underlying the development of CPP in female children remain unclear. Therefore, understanding the role of crucial metabolites may facilitate rapid diagnosis. Metabolomics is a powerful biological tool used to study disease phenotypes and plays an essential role in several aspects, such as biomarker discovery, the origin and development of a disease, and personalized treatment (Nicholson et al., 2005; Huang et al., 2013; Wishart, 2016). Therefore, physiological and pathological mechanisms underlying the development of diseases can be identified *via* metabolomic analysis.

At present, the first-line therapeutic agents for CPP are GnRH analogs (Bertelloni and Baroncelli, 2013), which inhibit gonadotropin secretion and negatively mediate GnRH receptors, leading to a reduction in the levels of gonadal steroids to prepubertal levels (Bertelloni et al., 2018). GnRH analogs can retard pubertal progression and bone maturation to ameliorate the development of an adult stature (Xue et al., 2018). However, GnRH analogs have side effects, such as local erythema, hyperlipidemia, central obesity, temporary vaginal bleeding, and the loss of bone density (Tonini and Lazzerini, 2000; Lee and Houk, 2006; Karamizadeh et al., 2013). Some medicinal herbs and prescriptions have been reported to treat PP (Yu et al., 2014; Lee et al., 2016). In this study, we used both a classical drug (injectable leuprorelin acetate microspheres) and the traditional Chinese medicinal formula Da-Bu-Yin (oral administration) to treat female children with PP. The drug dosage of injectable leuprorelin acetate microspheres was administered at 80 μg/kg every 28 days after the first injection of 120 μg/kg. The drug dosage of Da-Bu-Yin pilula was orally administered at 6 g twice daily. The combined treatment duration was 6 months. The serum metabolic profile of female children in the CPP (group S; *n* = 31 female children with CPP), treatment (group R; *n* = 29 female children treated with the two mentioned drugs), and healthy control (group N; *n* = 30 healthy female children) groups were investigated using UHPLC-Triple-TOF-MS. A total of six metabolites found in the treatment group were significantly correlated with short peptides (His-Arg-Lys-Glu and Lys-Met-His), PE [19:1(9Z)0:0], tumonoic acid I, palmitic amide, and linoleic acid–biotin. These metabolites regulate various pathways including aminoacyl–tRNA biosynthesis; phenylalanine, tyrosine, and tryptophan biosynthesis; and fatty acid biosynthesis. This study reveals new therapeutic metabolites based on metabolomic analyses. The key metabolites discovered may help develop approaches for rapid clinical diagnosis and individualized treatment of female children with CPP.

## 2. Materials and methods

### 2.1. Sample collection

All female children with CPP were recruited from The Fifth Affiliated Hospital of Harbin Medical University. The study was approved by the ethics committee of The Fifth Affiliated Hospital of Harbin Medical University (KY [2018] 002). CPP was diagnosed according to the 2015 edition of the Chinese guidelines for PP prevention and treatment. The detailed evaluation criteria are as follows: (1) early development of secondary sexual characteristics: The development of secondary sexual characteristics in girls and boys occurred before the ages of 8 and 9 years, respectively. (2) Linear growth acceleration: The annual growth rate was higher than that in healthy children. (3) Increased bone age: Bone age exceeded the actual age by ≥ 1 year. (4) Gonadal enlargement: Pelvic ultrasonography showed an increase in the female uterus and ovary volume and multiple follicles of a diameter of > 4 mm in the ovary; the testis volume was > 4 ml in boys. (5) The hypothalamic–pituitary–gonadal axis (HPGA) function was initiated, and the serum gonadotropin and sex hormones reached pubertal levels.

The healthy control group (group N; 30 female children) was formed based on the physical examination of female children. The disease group (group S; 31 female children) included female children with CPP. The initial diagnosis of the sera sample of the 31 female children was collected as a disease group. The treatment group was based on our available traceable cases (group R; 29 female children). A chemical drug (a GnRH analog) and a traditional Chinese medicinal formula were combined to treat female children with CPP in 6 months (group R). Non-targeted metabolomic analysis was performed to analyze differences in serum metabolites among the healthy control (group N), CPP (group S), and treatment (group R) groups. The GnRH analog used was injectable leuprorelin acetate under the trade name Bey [National Medical Products Administration (NMPA) ratification number: Guo Yao Zhun Zi-H20093852; lot number of the existing pharmaceutical administration: 210614]. The traditional Chinese medicinal formula used was DBYW (NMPA ratification number: Guo Yao Zhun Zi-Z20043458).

Sample information is summarized in Table 1.

**TABLE 1:** Information of study participants.

	Control (N)	CPP (S)	Treatment (R)	*P* (N vs. S)	Statistical difference (N vs. S)	*P* (S vs. R)	Statistical difference (S vs. R)	*P* (N vs. R)	Statistical difference (N vs. R)
Number	30	31	29						
Sex	Female	Female	Female						
Age (years)	8.90 ± 0.32	8.35 ± 0.28	8.62 ± 0.31	0.204		0.528		0.529	
LH	0.20 ± 0.01 (< 0.3)	1.34 ± 0.35 (> 0.3)	0.49 ± 0.17 (> 0.3)	<0.01 (0.002)	Yes	<0.05 (0.038)	Yes	0.076	No
LH/FSH	0.30 ± 0.02	0.44 ± 0.08	0.37 ± 0.06	0.075		0.459		0.303	
Left ovary volume (ml)	0.73 ± 0.03 (< 1)	2.41 ± 0.24 (> 1)	1.58 ± 0.21 (> 1)	<0.001	Yes	0.0023	Yes	<0.001	Yes
Right ovary volume (ml)	0.71 ± 0.03 (< 1)	2.51 ± 0.28 (> 1)	1.81 ± 0.18 (> 1)	<0.001	Yes	0.0451	Yes	<0.001	Yes
Left follicles (number)	2.33 ± 0.15 (< 4)	4.71 ± 0.28 (> 4)	3.48 ± 0.31 (< 4)	<0.001	Yes	0.0003	Yes	<0.001	Yes
Right follicles (number)	2.37 ± 0.15 (< 4)	4.87 ± 0.30 (> 4)	3.55 ± 0.35 (< 4)	<0.001	Yes	0.0002	Yes	<0.001	Yes
Left follicle size (mm)	<4	>4	<4						
Right follicle size (mm)	<4	>4	<4						
Uterus length (cm)	2.30 ± 0.09	2.58 ± 0.13	2.53 ± 0.17	0.079		0.814		0.232	
Bone age (years)	8.90 ± 0.32	9.48 ± 0.25	9.74 ± 0.31	0.153		0.518		0.061	
Bone age—age	0	>1	>1						

N, healthy female children (*n* = 30); S, female children with central precocious puberty (*n* = 31); R, female children treated with drugs used in this study (*n* = 29). All data are expressed as mean ± SE (two-tailed *t*-test).

The level of luteinizing hormone (LH) and follicle size (left and right) have returned to normal. In addition, the ovary volume decreased; however, it did not return to normal and at least showed a downward trend from the disease group.

### 2.2. Non-targeted metabolomic analysis

Chromatography was performed on an ultra-high-pressure liquid chromatography (UHPLC) system (Agilent 1290, USA). The chromatographic column used was ACQUITY UHPLC HSS T3 (1.8 μm; 2.1 × 100 mm) (Waters). Mobile phase A included 0.1% formic acid in the water, and mobile phase B included 0.1% formic acid in acetonitrile (Zhao et al., 2014). Gradient elution was performed as follows: 5% B for 1 min, which was changed linearly to 10% B within 1 min, which was subsequently changed linearly to 95% B within 12 min and held for 2 min, which was eventually changed linearly to 5% B within 1 min and held for 3 min. The analytical column and autosampler temperatures were 35°C and 4°C, respectively. The sample volume was 5 μl for each run, and the column eluent was directly analyzed on the MS system (Zhao et al., 2014).

For analysis, accurately 200 μl of serum was mixed with 4 volumes of methanol/acetonitrile (1:1, v/v). All samples were shaken for 30 s and subjected to ultrasonication for 10 min in ice-cold water. The samples were incubated at -20°C for 2 h to facilitate the precipitation of protein and were subsequently subjected to centrifugation at 13,000 × *g* for 15 min at 4°C. The serum supernatant was collected, dried under a vacuum, and subjected to centrifugation at 4°C. Subsequently, the aliquots were resuspended in 200 μl of acetonitrile/water (1:1), shaken for 30 s, and subjected to centrifugation at 13,000 × *g* for 15 min at 4°C, and the supernatants were collected. Approximately 150 μl of the supernatant was analyzed on a UHPLC system (Agilent 1290, USA) coupled with a Q-TOF system (Agilent 6545, USA). The remaining supernatants were mixed to prepare quality control (QC) samples. QC was performed after every 15 serum samples were analyzed.

Data were obtained using the auto MS/MS mode from 50 to 1,100 m/z. Collision energies for collision-induced dissociation were 20 and 40 V. MS parameters were set as follows: ion source dry gas temperature, 320°C; N_2_ gas flow, 8 L/min; sheath gas temperature, 350°C; sheath gas flow, 12 L/min; and ion spray voltage, 4,000 (positive ion) and 3,500 V (negative ion).

### 2.3. Data collection and analysis

Data files from the Q-TOF-MS system were converted to the .abf format using the Analysis Base File Converter software. Peak detection, chromatogram deconvolution, and other data analyses were performed using the MSDIAL3.82 software with the following parameters: alignment-MS1 tolerance, 0.01 Da; retention time tolerance, 0.2 min; identification accurate mass tolerance (MS1), 0.005 Da; MS2, 0.05 Da; and identification cutoff score, 60%. The HMDB,^1^ METLIN,^2^ Massbank,^3^ and KEGG^4^ databases were used to identify key metabolites and metabolic pathways.

**FIGURE 1:**
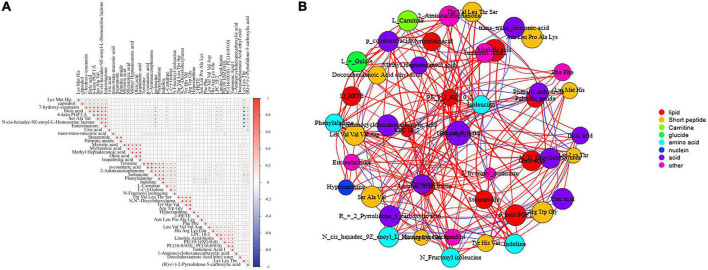
**(A)** Correlation analysis of the identified metabolites. Positive correlations are indicated in red, whereas negative correlations are indicated in blue. **(B)** Network analysis of the identified metabolites. Positive correlations are indicated in red, whereas negative correlations are indicated in blue (Spearman’s correlation coefficient threshold = 0.4).

Multivariate statistical analysis was performed using the SIMCA-P software (version 14.1; Umetrics, Umea, Sweden). The matrix was imported into R to observe the overall distribution of samples and assess the stability of the whole process using principal component analysis (PCA). Partial least squares discriminant analysis (PLS-DA) and orthogonal partial least squares discriminant analysis (OPLS-DA) were used to distinguish metabolites between groups. To evaluate the quality of the model, sevenfold cross-validation and 200 permutation tests [response permutation testing (RPT)] were performed. The permutation test was used to assess the validity and degree of overfitting of the model. VIP values of > 1.0 and *p*-values of < 0.05 indicated significant differences. The Mann–Whitney U test was used to compare two groups, with *p*-values of < 0.05 indicating significant differences. The false discovery rate (FDR) was used for comparing multiple groups (*p* < 0.10). The ratios of different metabolites between the average of those in the healthy control group and the two experimental groups were calculated. Figure 1A is created using the hiplot online website^5^ and Figure 1B using Wekemo Bioincloud.^6^

## 3. Results

### 3.1. Metabolomic analysis of the drug treatment of CPP *via* UHPLC-TOF-MS

**FIGURE 2:**
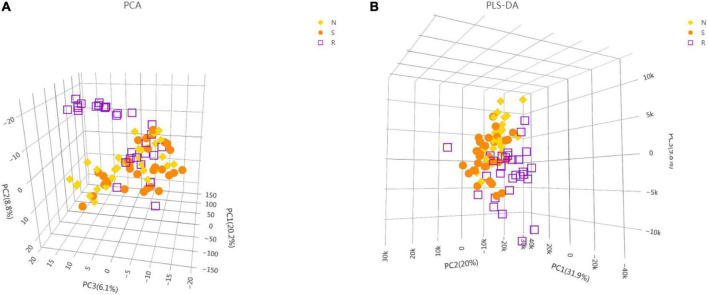
Metabolic profiling analysis of the serum of female children with central precocious puberty. **(A)** Score plot generated using the 3D-PCA model. **(B)** Score plot generated using the 3D-PLS-DA model (Supplementary Figure 1A). OPLS-DA between the N and S groups (Supplementary Figure 1B). OPLS-DA between the R and S groups (Supplementary Figure 1C). OPLS-DA between the R and N groups (Supplementary Figure 1D). Qualitative analysis of the OPLS-DA model (N vs. S) *via* response permutation testing (RPT) (Supplementary Figure 1E). Qualitative analysis of the OPLS-DA model (R vs. S) *via* RPT (Supplementary Figure 1F). Qualitative analysis of the OPLS-DA model (R vs. N) *via* RPT. N, healthy female children (*n* = 30); S, female children with central precocious puberty (*n* = 31); R, female children treated with drugs used in this study (*n* = 29).

To identify crucial metabolites associated with the treatment of CPP, the serum metabolic profiles of female children in the three groups (N, S, and R groups) were analyzed *via* UHPLC-TOF-MS. Data analyzed using the SIMCA-P software, 3D-PCA, and PLS-DA are demonstrated in Figures 2A, B. OPLS-DA was also used to screen for critical metabolites in each group (Supplementary Figures 1A–C). Differences in metabolites were analyzed between the S and N, R and N, and R and S groups. All OPLS-DA models were reliable because the permutation tests did not reveal overfitting (Supplementary Figures 1D–F). Several critical metabolites and metabolic pathways associated with treatment were identified in the serum of female children (Supplementary Table 1).

### 3.2. Visualization of differentially expressed metabolites using volcano plots

**FIGURE 3:**
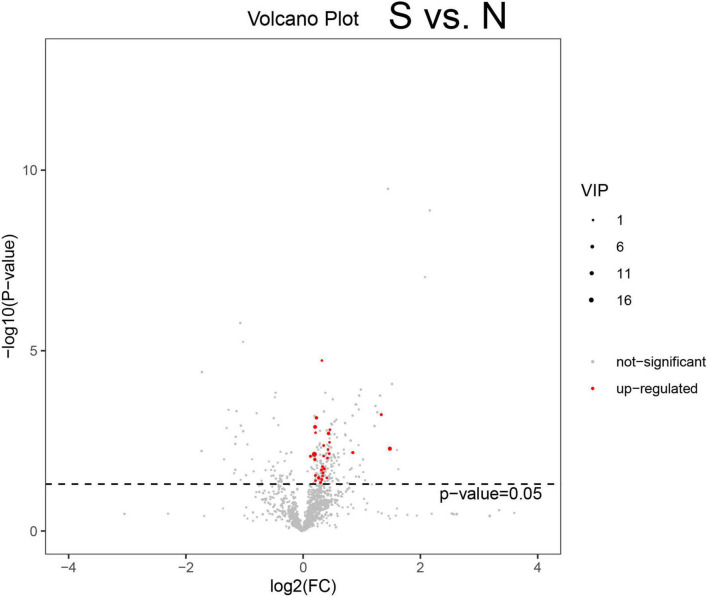
Volcano plot demonstrating differentially expressed metabolites. A represents metabolites that are downregulated, not significant, or upregulated in different groups of female children. The abscissa is *log*_2_(*FC*), the left ordinate is −*log*_10_(*p*−*value*),and the right ordinate is VIP (variable importance in projection). Volcano plot demonstrating differentially expressed metabolites between the S and N groups (Supplementary Figure 2A). Volcano plot demonstrating differentially expressed metabolites between the R and S groups (Supplementary Figure 2B). Volcano plot demonstrating differentially expressed metabolites between the R and N groups. N, healthy female children (*n* = 30); S, female children with central precocious puberty (*n* = 31); R, female children treated with drugs used in this study (*n* = 29).

Differentially expressed metabolites were analyzed among the N, S, and R groups (Figure 2A and Supplementary Figures 2A, B). A total of 21 metabolites were found to be upregulated in the S group compared with the N group (Figure 3A). In addition, a total of 28 downregulated and 21 upregulated metabolites were identified in the R group (Supplementary Figure 2A). A total of 55 differentially expressed metabolites were identified between the R and N groups (Supplementary Figure 2B), including 24 downregulated and 31 upregulated metabolites. These findings reveal that some specific metabolites play a significant role in the treatment of CPP.

### 3.3. Metabolite–metabolite correlation analysis

Pearson’s correlation analysis was performed to examine the correlation among the identified metabolites in female children with CPP. Figure 1A shows the correlation among the top 50 differentially expressed metabolites. Lys-Met-His, one of the oligopeptides, had a significant positive correlation with ilicic acid, 7-hydroxy-coumarin, 6-keto PGF1α, and *N*-cis-hexadec-9Z-enoyl-L-homoserine lactone. Figure 1B shows the network analysis of the 46 identified metabolites in female children with CPP. The identified metabolites were classified as lipids, short peptides, carnitines, glucides, amino acids, nuclein, acids, and others.

### 3.4. KEGG pathway enrichment analysis

**FIGURE 4:**
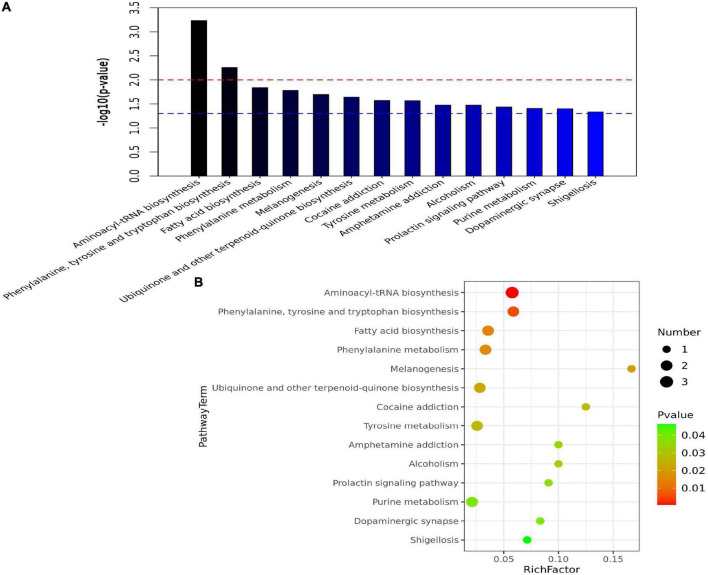
Pathway enrichment analysis. **(A)** Bar charts of enriched pathways among the R, S, and N groups. The abscissa is the pathway, and the ordinate is −*log*_10_(*p*−*value*). The blue line indicates a *p*-value threshold of 0.05, and the red line indicates a *p*-value threshold of 0.01. **(B)** Bubble chart of enriched pathways among the R, S, and N groups. The abscissa is the rich factor (the detailed description is provided in section “3.4. KEGG pathway enrichment analysis” of results), and the left ordinate is the pathway.

A total of 14 pathways (including aminoacyl–tRNA biosynthesis; phenylalanine, tyrosine, and tryptophan biosynthesis; and fatty acid biosynthesis) were significantly enriched in the three groups (Figure 4). As shown in Figure 1A, phenylalanine was positively correlated with Phe-Phe, hypoxanthine, (R)-(+)-2-pyrrolidone-5-carboxylic acid, Lys-Lys-Thr, 12-HETE, and Asn-Leu-Pro-Ala-Lys but was negatively correlated with trans-trans-muconic acid, 6-keto PGF1α, and desalkyl verapamil D617. Tyrosine was positively correlated with 2-aminoacetophenone, p-coumaric acid, indoline, phenylalanine, and isoleucine but was negatively correlated with *N*-cis-hexadec-9Z-enoyl-L-homoserine lactone, 6-keto PGF1α, and trans-trans-muconic acid.

As shown in Figures 4A, B, aminoacyl–tRNA biosynthesis was the most significantly enriched pathway in the R group. In Figure 4B, the abscissa indicates the rich factor, which was calculated by dividing the number of differentially expressed metabolites in the corresponding metabolic pathway by the number of total metabolites identified in the pathway. The higher the rich factor, the larger the degree of pathway enrichment. Colors are expressed on a green to red linear scale with gradually decreasing *p*-values. The bigger the bubble, the more the number of metabolites in the pathway.

### 3.5. Short peptides

**FIGURE 5:**
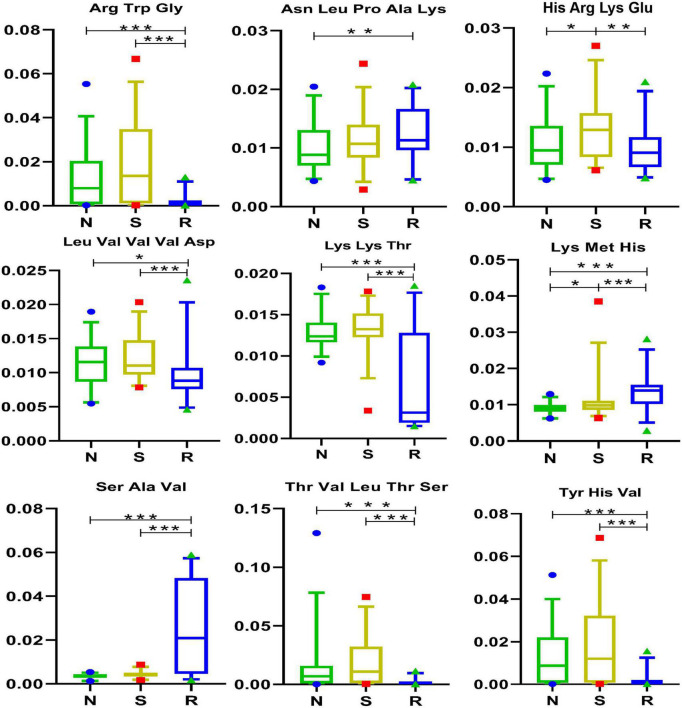
Short peptides in box and-whisker plots. N, healthy female children (*n* = 30); S, female children with central precocious puberty (*n* = 31); R, female children treated with drugs used in this study (*n* = 29). Plot are expressed as the 5th–95th percentiles (*p < 0.05; **p < 0.01; ***p < 0.001; two-tailed Mann–Whitney *U*-test).

Short peptides contain fundamental biological information and are a precursor to life. Scholars have recently developed a greater interest in short peptides owing to their unique features and prospects for developing innovative bio-therapies (Apostolopoulos et al., 2021). In this study, two short peptides, namely, His-Arg-Lys-Glu and Lys-Met-His, were differentially expressed between the N and S groups (Figure 5). In addition, eight short peptides, namely, Arg-Trp-Glu, His-Arg-Lys-Glu, Leu-Val-Val-Val-Asp, Lys-Lys-Thr, Lys-Met-His, Ser-Ala-Val, Thr-Val-Leu-Thr-Ser, and Tyr-His-Val, were differentially expressed between the R and S groups. These peptides were upregulated after drug treatment, except for Lys-Met-His and Ser-Ala-Val.

Furthermore, eight short peptides, namely, Arg-Trp-Glu, Asn-Leu-Pro-Ala-Lys, Leu-Val-Val-Val-Asp, Lys-Lys-Thr, Lys-Met-His, Ser-Ala-Val, Thr-Val-Leu-Thr-Ser, and Tyr-His-Val were differentially expressed between the N and R groups. The short peptides His-Arg-Lys-Glu and Lys-Met-His may play a significant role in CPP among female children and may be potential targets for the treatment of CPP. Furthermore, Lys-Met-His was positively correlated with endiline, ilicic acid, 7-hydroxy-coumarin, desalkyl verapamil D617, 6-keto-PGF1α, enterolactone, and *N*-cis-hexadec-9Z-enoyl-L-homoserine lactone (Figure 1A) but was negatively correlated with Lys-Lys-Thr, isoleucine, Asn-Leu-Pro-Ala-Lys, capsidiol, and myristoleic acid.

### 3.6. Amino acids and lipids

**FIGURE 6:**
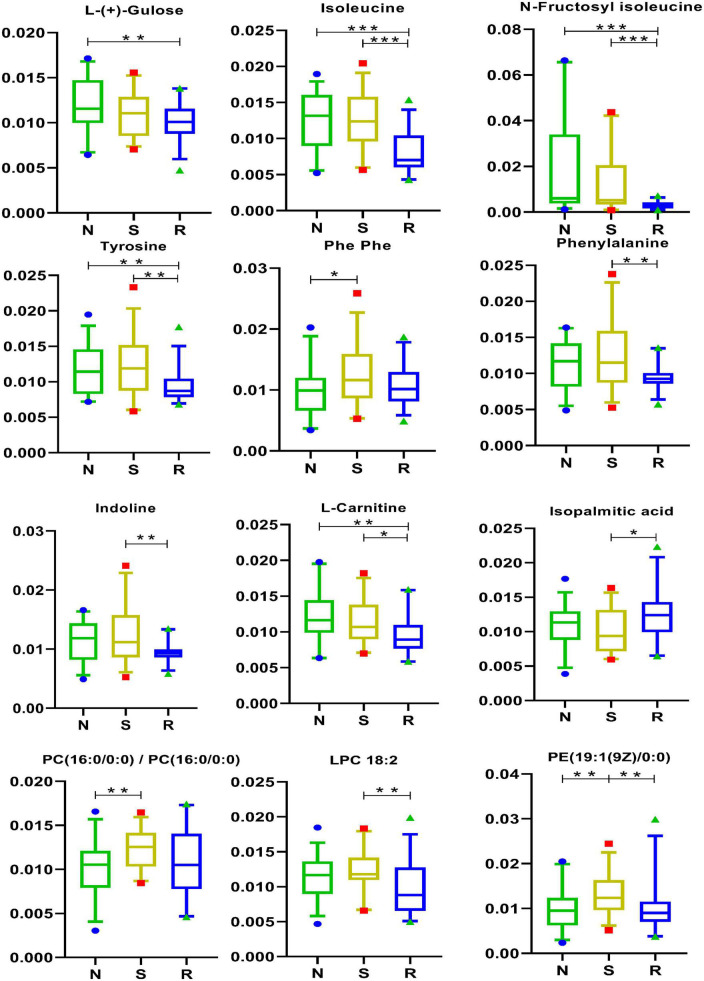
Amino acid pathways and lipids in box-and-whisker plots. N, healthy female children (*n* = 30); S, female children with central precocious puberty (*n* = 31); R, female children treated with drugs used in this study (*n* = 29). Plot are expressed as the 5th–95th percentiles (*p < 0.05; **p < 0.01; ***p < 0.001; two-tailed Mann–Whitney *U*-test).

As shown in Figure 6, the expression of 5 amino acids, namely, isoleucine, *N*-fructosyl isoleucine, indoline, phenylalanine, and tyrosine, was lower in the R group than that in the S group. In addition, the expression of L-(+)-gulose, isoleucine, *N*-fructosyl isoleucine, and tyrosine was lower in the R and N groups. As shown in Figure 6, the drug may act by downregulating the above-mentioned five amino acids, which are closely involved in the modulation of CPP among female children.

The results of the analysis of lipid metabolites are shown in Figure 6. As shown in the figure, the drug may act by regulating PE [19:1(9Z)0:0]. As shown in Figure 1A, PE [19:1(9Z)0:0] had a positive correlation with three metabolites [tumonoic acid I, PC (16:0/0:0), and Phe-Phe] and a negative correlation with one metabolite (trans-trans-muconic acid).

### 3.7. Acid metabolite analysis

**FIGURE 7:**
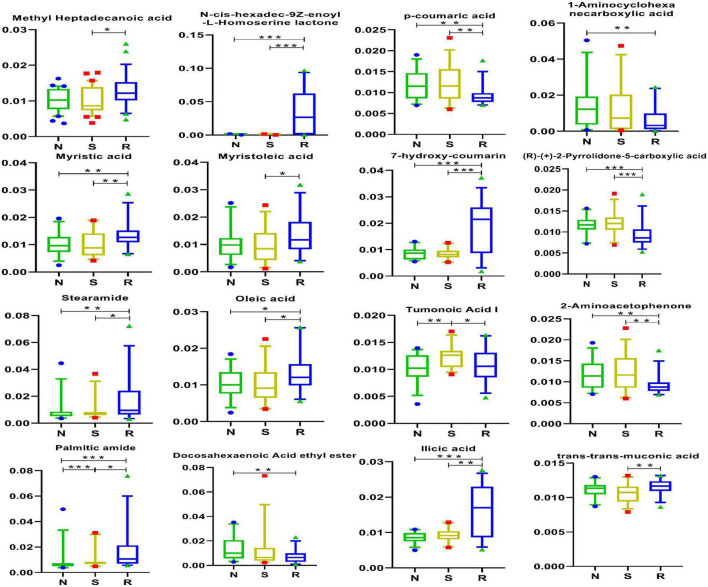
Acid metabolites in box-and-whisker plots. N, healthy female children (*n* = 30); S, female children with central precocious puberty (*n* = 31); R, female children treated with drugs used in this study (*n* = 29). Plot are expressed as the 5th–95th percentiles (*p < 0.05; **p < 0.01; ***p < 0.001; two-tailed Mann–Whitney *U*-test).

Differentially expressed acid metabolites were identified to distinguish among female children in the three groups. As shown in Figure 7, tumonoic acid I and palmitic amide may be associated with the pathogenesis of CPP. The expression of tumonoic acid I was increased and that of phthalic anhydride was decreased in the CPP group; however, these changes were reversed after drug treatment.

As shown in Figure 1A, tumonoic acid I showed a significant positive correlation with Phe-Phe, 12-HETE, hypoxanthine, and Lys-Lys-Thr but a negative correlation with trans-trans-muconic acid, ilicic acid, and 6-keto PGF1α. In addition, palmitic amide was positively correlated with stearamide, oleic acid, and Lys-Met-His.

### 3.8. Arachidonic acid and other metabolites

**FIGURE 8:**
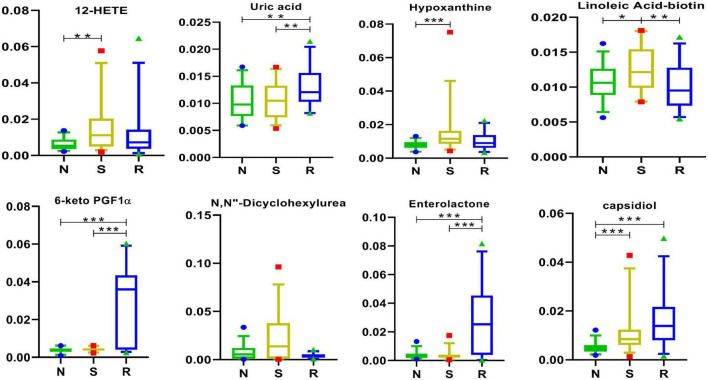
Arachidonic acid and other metabolites in box-and-whisker plots. N, healthy female children (*n* = 30); S, female children with central precocious puberty (*n* = 31); R, female children treated with drugs used in this study (*n* = 29). Plots are expressed as the 5th–95th percentiles (*p < 0.05; **p < 0.01; ***p < 0.001; two-tailed Mann–Whitney *U*-test).

It remains unclear whether one or more biomarkers are involved in certain physiological processes or pathological alterations in CPP; however, linoleic acid–biotin can be one of them in Figure 8. In this study, the expression of linoleic acid–biotin was higher in the S group than that in the N group but was decreased after drug treatment. In addition, linoleic acid–biotin was positively correlated with PE [19:1(9Z)/0:0], PC (16:0/0:0), tumonoic acid I, isoleucine, and Lys-Lys-Thr and was negatively correlated with uric acid, ilicic acid, 7-hydroxy-coumarin, desalkyl verapamil D617, 6-keto PGF1α, and *N*-cis-hexadec-9Z-enoyl-L-homoserine lactone (Figure 1A).

The expression of 12-HETE, hypoxanthine, and capsidiol was different between the S and N groups.

### 3.9. ROC curves and AUC analysis

**FIGURE 9:**
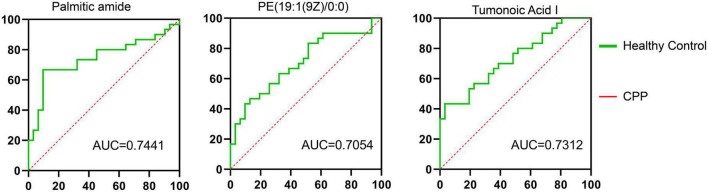
Serum levels and ROC analysis of altered metabolites. Healthy female children, *n* = 30; female children with central precocious puberty (CPP), *n* = 31.

Figures 1, 3, 4–8 of the results provided significant insights into drug therapy mechanisms and personalized treatment, but we also analyzed ROC curves for metabolites. The correlation between the levels of palmitic amide, PE [19:1(9Z)/0:0], and tumonoic acid I and between the treatment response and progression of CPP among children was analyzed (Figure 9 and Supplementary Table 2). The AUC values of the three metabolites were 0.7441, 0.7054, and 0.7312, respectively.

## 4. Discussion

The incidence of CPP among girls has increased in recent years. However, the mechanisms underlying the pathogenic processes of CPP remain unclear. Identification of key metabolites may help understand these underlying mechanisms and develop approaches for prompt diagnosis, which is necessary for the effective treatment of CPP. In this study, we identified key metabolites and pathways associated with the treatment of CPP *via* UHPLC-MS-MS.

### 4.1. Key metabolites

His-Arg-Lys-Glu, Lys-Met-His, PE [19:1(9Z)0:0], tumonoic acid I, palmitic amide, and linoleic acid–biotin may play a significant role in CPP among female children.

Several short peptides (2–20 amino acids) are partially obtained *via* enzymatic hydrolysis of protein and are important bioactive peptides (Zhang et al., 2019). In recent years, short peptides have been of considerable interest in the branch of biology, chemistry, and medicine owing to their unique structural and functional diversity (Apostolopoulos et al., 2021). Short peptides play an important role in the immune system and are responsible for transmitting most immunological information (Parhiz et al., 2013). Studies have reported that short peptides play an important role in many diseases, including neurodegenerative diseases (Jang et al., 2016), Alzheimer’s disease (Russell-Aulet et al., 1999; Frutos et al., 2007; Tatman et al., 2016; Darawshe et al., 2019), and rheumatoid arthritis (Darawshe et al., 2019), and have therapeutic effects (New et al., 2014; Tatman et al., 2016; Baig et al., 2018; Horsley et al., 2020). In this study, two short peptides were differentially expressed between the N and S groups, whereas eight short peptides were differentially expressed between the R and S groups (Figure 6). These findings warrant further investigation and may serve as a reference for developing new therapeutics for CPP in female children.

Furthermore, the expression of PE [19:1(9Z)0:0] was higher in female children with CPP than in healthy female children but was decreased after drug treatment. PE [19:1(9Z)0:0], a phosphatidylethanolamine, is a brain phospholipid that plays an important role in improving reflex development, cognition, and memory (Müller et al., 2015; Li et al., 2016; Kim et al., 2019). As a breakdown product of PE, lysophosphatidylethanolamine (LPE) is present in the cells of all organisms. Alternatively, the perturbations of LPE may reflect PE dysfunction, which may be related to changes in the structural membrane and permeability of the plasma membrane (Gao et al., 2017).

The specific effects of tumonoic acid I, which is also known as (S)-1-[(E)-(2R,3S)-3-hydroxy-2,4-dimethyl-dodec-4-enoyl]-pyrrolidine-2-carboxylic acid, remain unclear. However, the parent nucleus in its chemical structure is pyrrolidine-2-carboxylic acid, which acts as an ionotropic glutamate receptor for some metabolites (Kayser et al., 2020). In the early phase of puberty, the hypothalamus, pituitary, and gonad axis are re-activated (Abreu and Kaiser, 2016), which is controlled by several neuroendocrine and metabolic factors (Walvoord, 2010; Plant, 2015). In addition, the GnRH pulse source is under both excitatory and inhibitory control, so, at the onset of puberty, the excitatory signal increases while the inhibitory signal decreases (Uenoyama et al., 2014). One neurotransmitter that stimulates GnRH pulse generation is glutamate (Farello et al., 2019). The pathogenesis of CPP has recently been linked to three genes: KISS1 (Silveira et al., 2010) encoding kisspeptin, its KISS1R receptor (Teles et al., 2008), and MKRN3, a gene deemed to act as a hypothalamic repressor on the gonadal axis (Farello et al., 2019). However, the exact mechanism of metabolites and genes remains to be studied and verified. A parent nucleus metabolite can decrease NH_3_ production by inhibiting the deamination of amino acids (Floret et al., 1999). In addition, some complexes not only exhibit marginal antimicrobial activity but also have good cytotoxic activity (Aiyelabola et al., 2017). Therefore, the role of tumonoic acid I in CPP warrants further investigation.

Palmitic amide is a primary fatty acid amide derived from palmitic acid (C16:0) (Whipple et al., 2021). Its plasma levels have been reported to increase with increasing age among patients who are overweight 2 (Guo et al., 2021). Additionally, palmitic amide promotes infection by enhancing host glycolysis (Zhang et al., 2021). As shown in Figure 3A, PE [19:1(9Z)0:0] was positively correlated with tumonoic acid I, PC (16:0/0:0), and Phe-Phe but was negatively correlated with trans-trans-muconic acid.

Linoleic acid–biotin is the combination of linoleic acid and biotin. As a precursor of arachidonic acid (Castrejón-Téllez et al., 2019), linoleic acid is an essential omega-6 (or n-6) polyunsaturated fatty acid (PUFA) (Vannice and Rasmussen, 2014; Jandacek, 2017). A recent review indicated that inflammatory effects can be decreased by reducing the intake of linoleic acid (Naughton et al., 2016). In addition, linoleic acid can promote tumor growth in some species and under some conditions (Jandacek, 2017).

Water-soluble biotin is required for normal cellular functions, growth, and development (Said, 2009). Biotin plays diverse roles in normal immune function (áez-Saldaña et al., 1998) and cell proliferation (Manthey et al., 2002). Therefore, its deficiency can lead to various clinical abnormalities, including growth retardation, neurological disorders, and dermatological abnormalities (León-Del-Río, 2019).

### 4.2. Key metabolic pathways

This study revealed 14 pathways (the top two pathways are aminoacyl–tRNA biosynthesis and phenylalanine, tyrosine, and tryptophan biosynthesis) significantly associated with CPP. The enrichment scores of the aminoacyl–tRNA biosynthesis pathway were the highest, suggesting its involvement in the occurrence and/or progression of CPP. In a study on the complexity of metabolic changes and nutrient remobilization during leaf senescence of tobacco, dynamic changes in the biosynthesis of aromatic amino acids, such as phenylalanine, tyrosine, and tryptophan, have been observed (Li et al., 2017). Therefore, an in-depth investigation should be performed to identify metabolic pathways underlying the pathogenesis of CPP and associated with its treatment.

### 4.3. Limitations

This study has some limitations. The age of female children in all groups was similar, and the associated diseases (except for injuries), dietary supplements, and diet differences were not examined. Furthermore, the number of samples used for screening key metabolites was relatively small (healthy female children, *n* = 30; patients with CPP, *n* = 31; patients treated with two drugs, *n* = 29) but met the requirements for human metabolomic analysis. However, the results should be validated in independent, large sample studies in the future. Moreover, female children with CPP can be primarily diagnosed based on the early appearance of secondary sexual characteristics, advanced bone age, gonadal enlargement and pubertal levels of serum gonadotropin, and sex hormones. This study demonstrated complementary methods that may improve clinical prediction and help understand the progression of CPP in female children.

## 5. Conclusion

A total of six metabolites were identified *via* metabolomic analysis, namely, His-Arg-Lys-Glu, Lys-Met-His, PE [19:1(9Z)0:0], tumonoic acid I, palmitic amide, and linoleic acid–biotin. These metabolites may play a significant role in the development of CPP. The expression of six metabolites was higher in female children with CPP than in healthy female children or those treated with the two drugs used in the study. In addition, a total of 14 pathways were significantly associated with the treatment of CPP. These findings enhance the understanding of molecular processes underlying CPP and may provide potential clues for the treatment of female children with CPP.

## Data availability statement

The original contributions presented in this study are included in the article/Supplementary material, further inquiries can be directed to the corresponding authors.

## Ethics statement

The Ethics Committee approved the study of the Fifth Affiliated Hospital of Harbin Medical University [KY (2018) 002]. Written informed consent to participate in this study was provided by the participants’ legal guardian/next of kin. Written informed consent was obtained from the individual(s), and minor(s)’ legal guardian/next of kin, for the publication of any potentially identifiable images or data included in this article.

## Author contributions

D-JY, Z-CX, and G-YC designed the project and provided funding. G-YC wrote the manuscript. YC, Y-SJ, L-QW, L-LW, J-YS, CL, and H-LT performed the sample collection. G-YC and D-JY performed sample testing. G-YC and QL carried out figures. Z-CX and J-YS performed the data interpretation. G-YC, L-ZW, J-CL, and Z-CX performed the data analysis. All authors involved in editing the manuscript.

## Footnotes

1
http://www.hmdb.ca/

2
http://metlin.scripps.edu/

3
http://www.massbank.jp

4
https://www.genome.jp/

5
https://hiplot-academic.com

6
https://www.bioincloud.tech

## Supplementary material

Supplementary Figure 1 OPLS-DA between the N and S groups.

Supplementary Figure 2 OPLS-DA between the R and S groups.

Supplementary Figure 3 OPLS-DA between the R and N groups.

Supplementary Figure 4 Qualitative analysis of the OPLS-DA model (N vs. S) *via* response permutation testing (RPT).

Supplementary Figure 5 Qualitative analysis of the OPLS-DA model (R vs. S) *via* RPT.

Supplementary Figure 6 Qualitative analysis of the OPLS-DA model (R vs. N) *via* RPT.

Supplementary Figure 7 Volcano plot demonstrating differentially expressed metabolites between the R and S groups.

Supplementary Figure 8 Volcano plot demonstrating differentially expressed metabolites between the R and N groups.

Supplementary Table 1 Data of key differential metabolites.

Supplementary Table 2 Data of ROC analysis.
