# Supplementary figures and images for: Serum metabolomic analysis reveals key metabolites in drug treatment of central precocious puberty in female children
Source: Front Mol Neurosci. 2023 Jan 27;15:972297. doi: 10.3389/fnmol.2022.972297 (PMC9912178; doi:10.3389/fnmol.2022.972297)

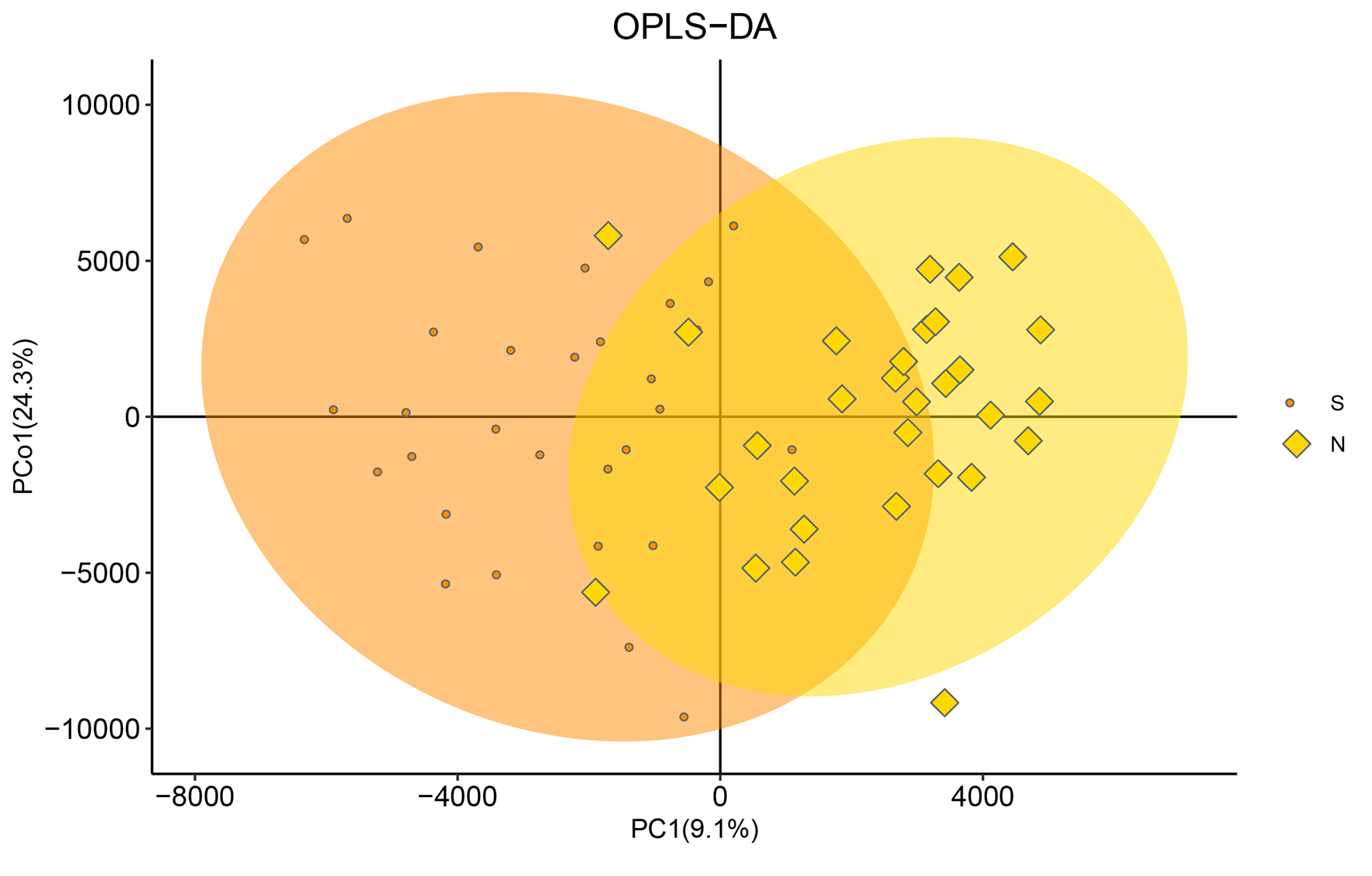

Supplement: Supplementary Figure 1 — OPLS-DA between the N and S groups. [file Image_1.tif]

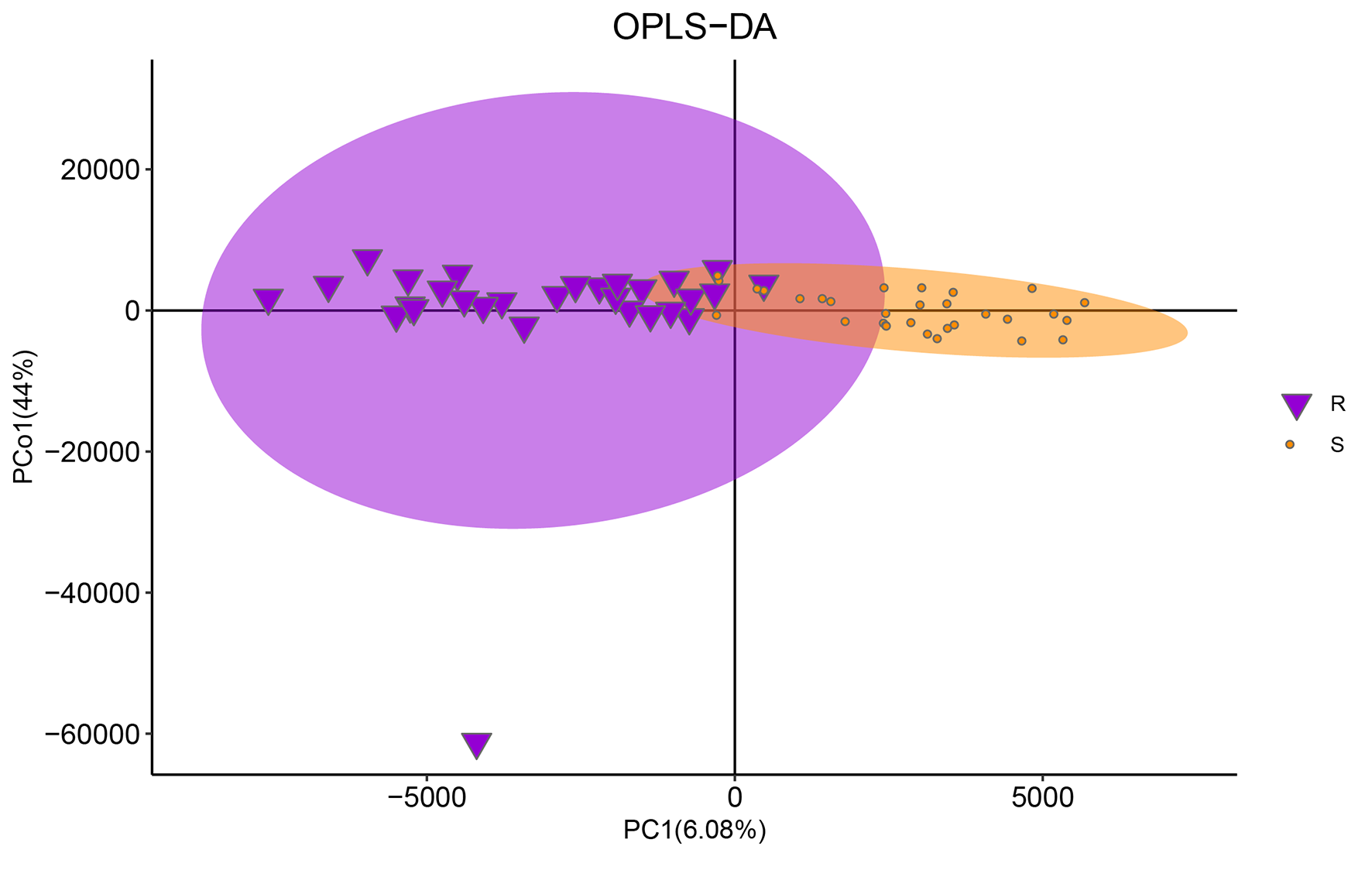

Supplement: Supplementary Figure 2 — OPLS-DA between the R and S groups. [file Image_2.tif]

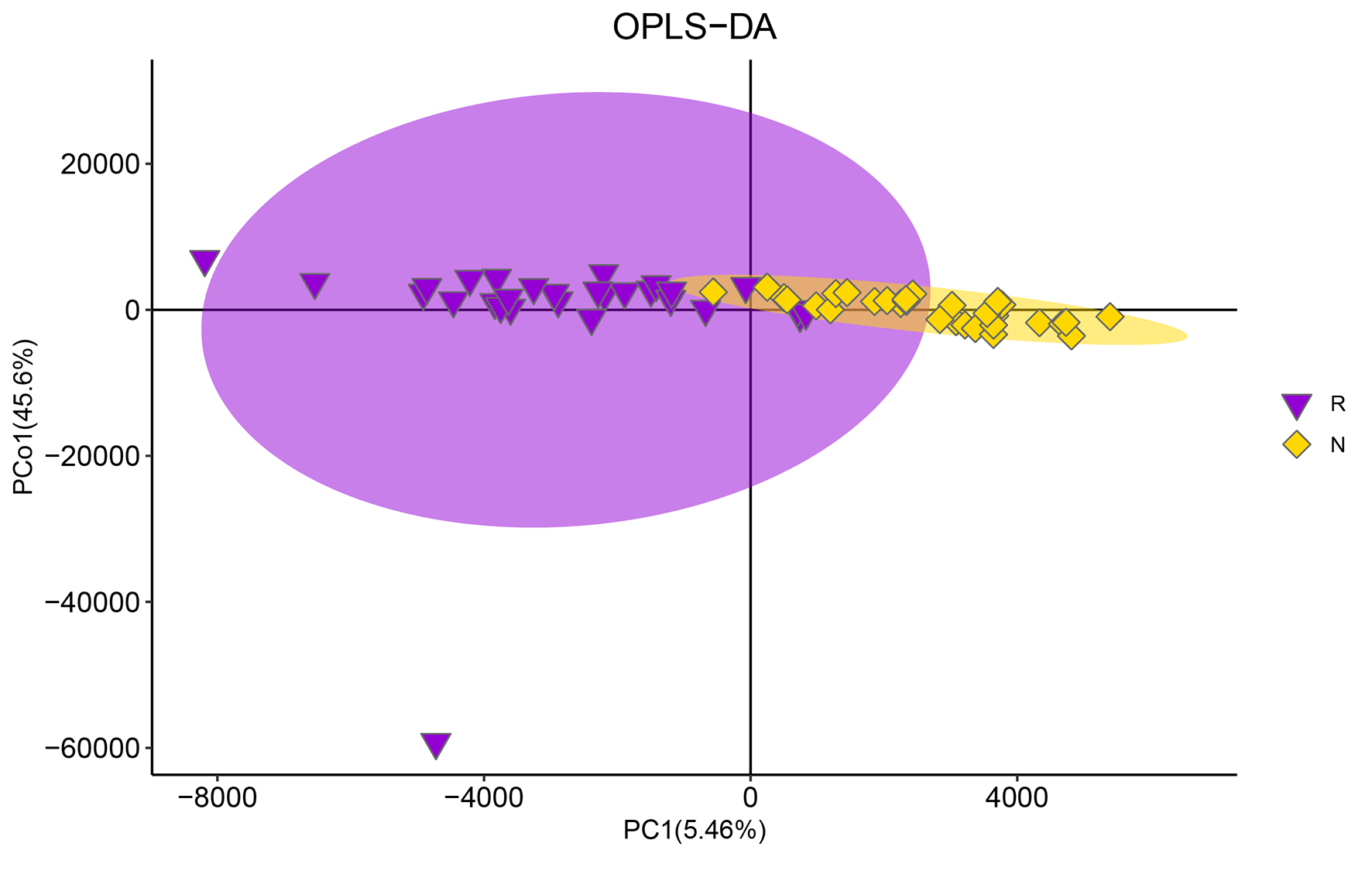

Supplement: Supplementary Figure 3 — OPLS-DA between the R and N groups. [file Image_3.tif]

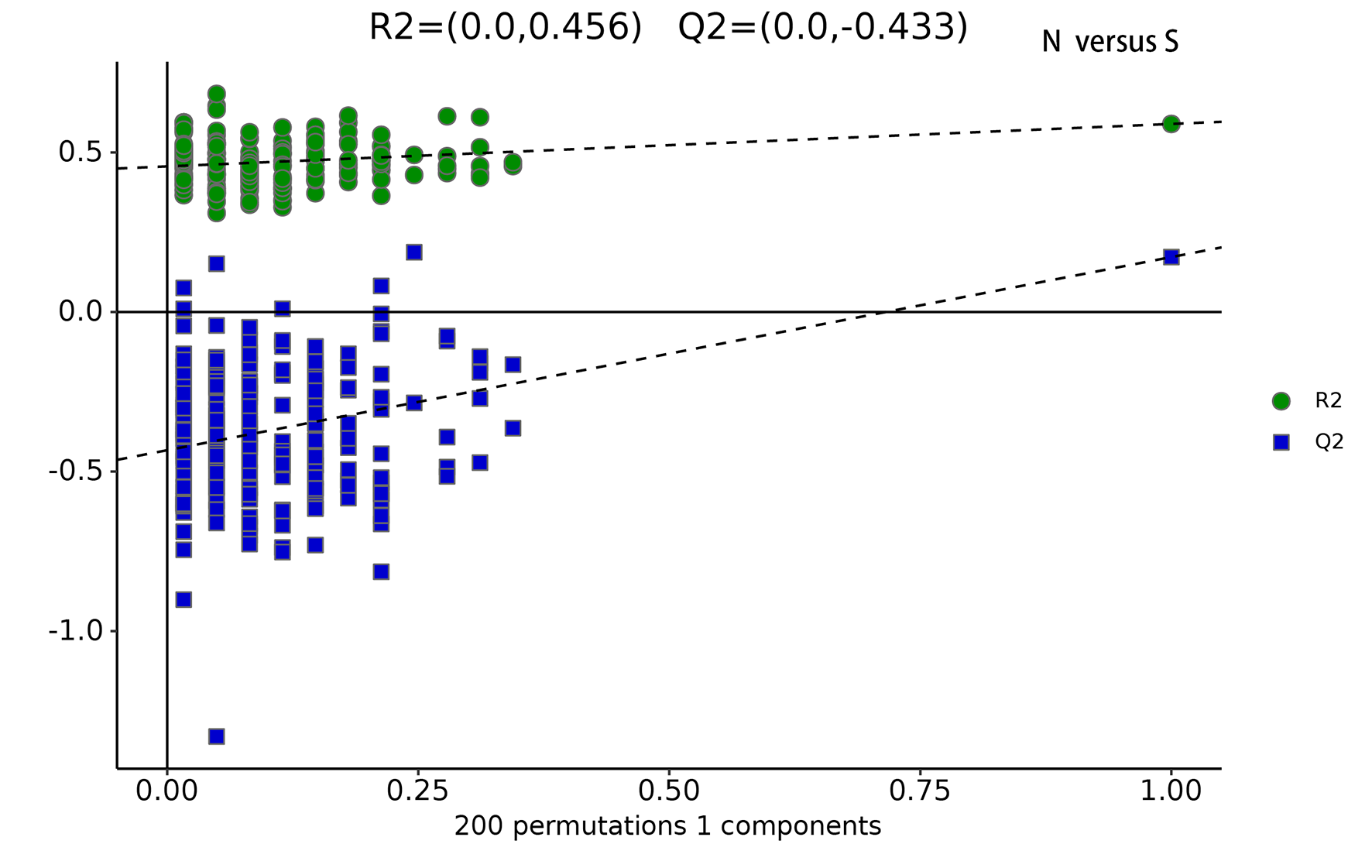

Supplement: Supplementary Figure 4 — Qualitative analysis of the OPLS-DA model (N vs. S) via response permutation testing (RPT). [file Image_4.tif]

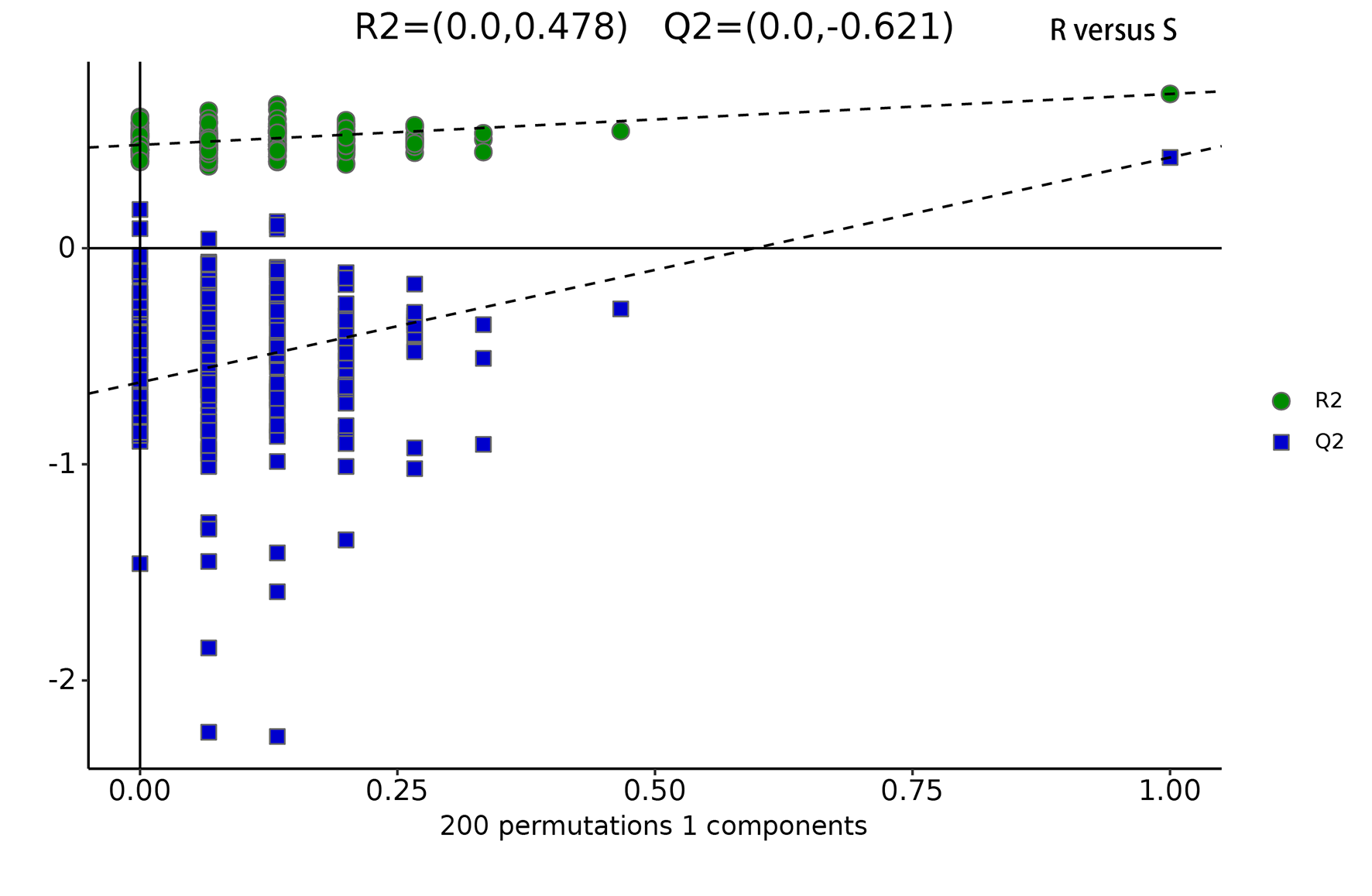

Supplement: Supplementary Figure 5 — Qualitative analysis of the OPLS-DA model (R vs. S) via RPT. [file Image_5.tif]

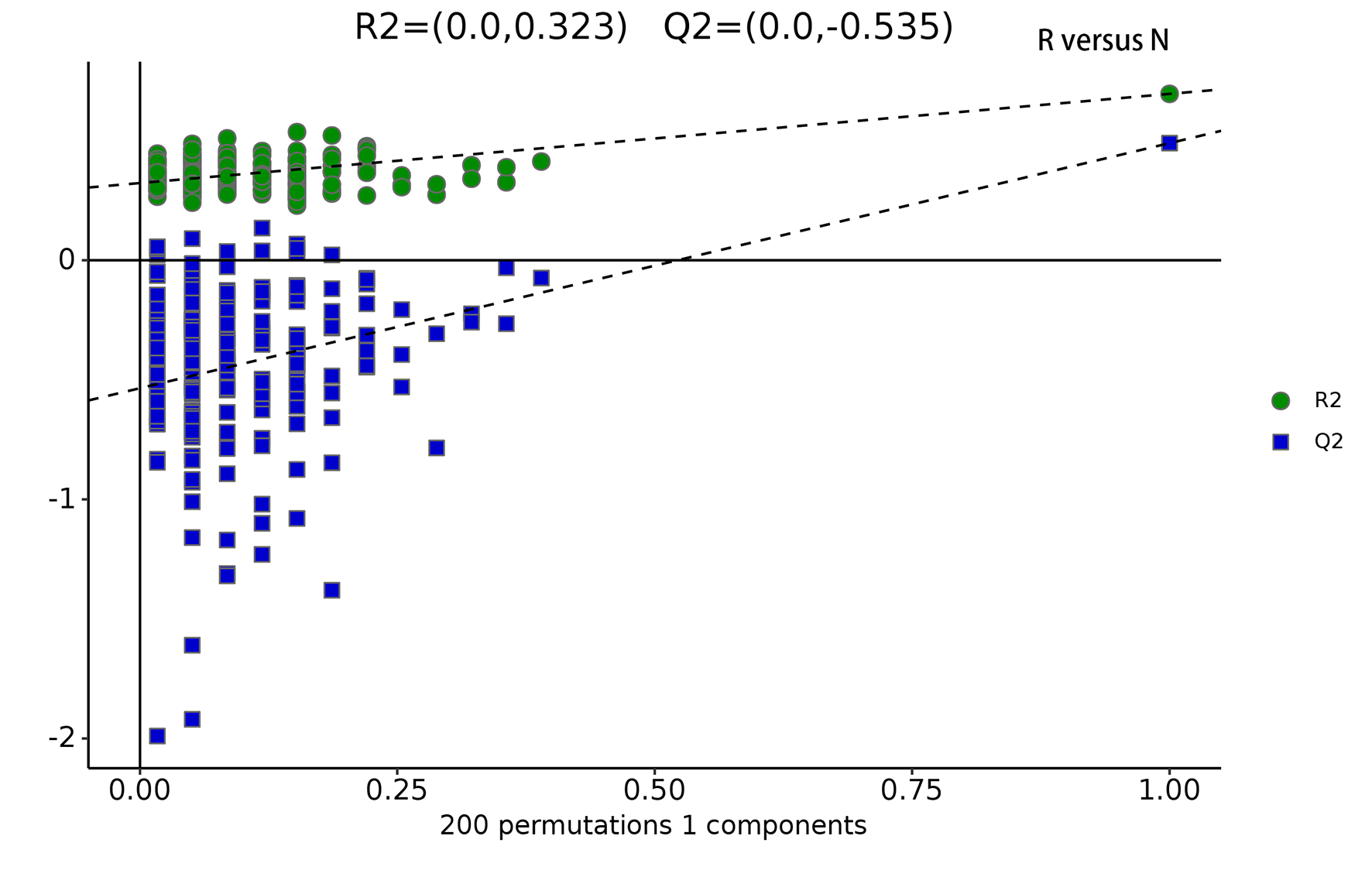

Supplement: Supplementary Figure 6 — Qualitative analysis of the OPLS-DA model (R vs. N) via RPT. [file Image_6.tif]

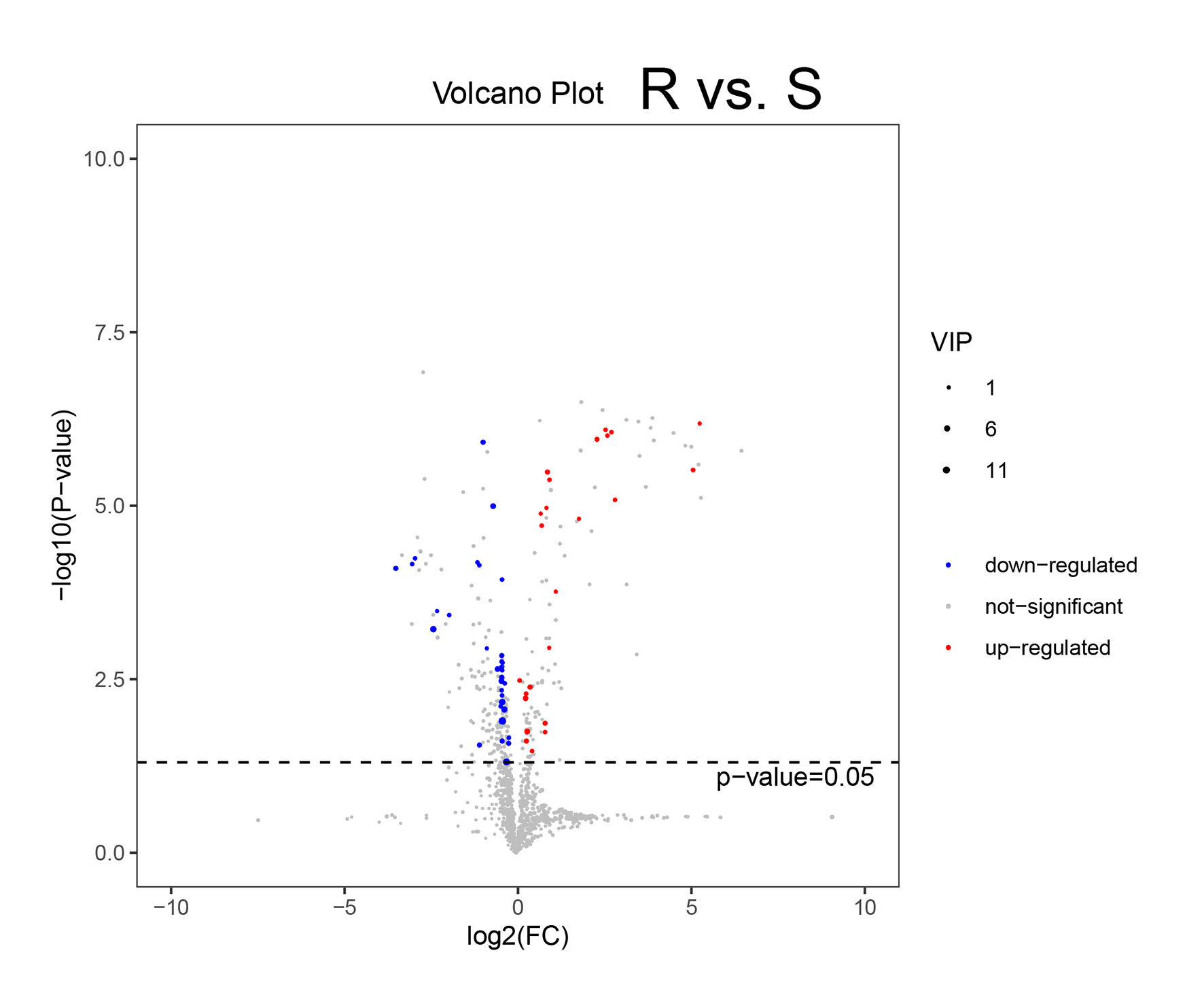

Supplement: Supplementary Figure 7 — Volcano plot demonstrating differentially expressed metabolites between the R and S groups. [file Image_7.tif]

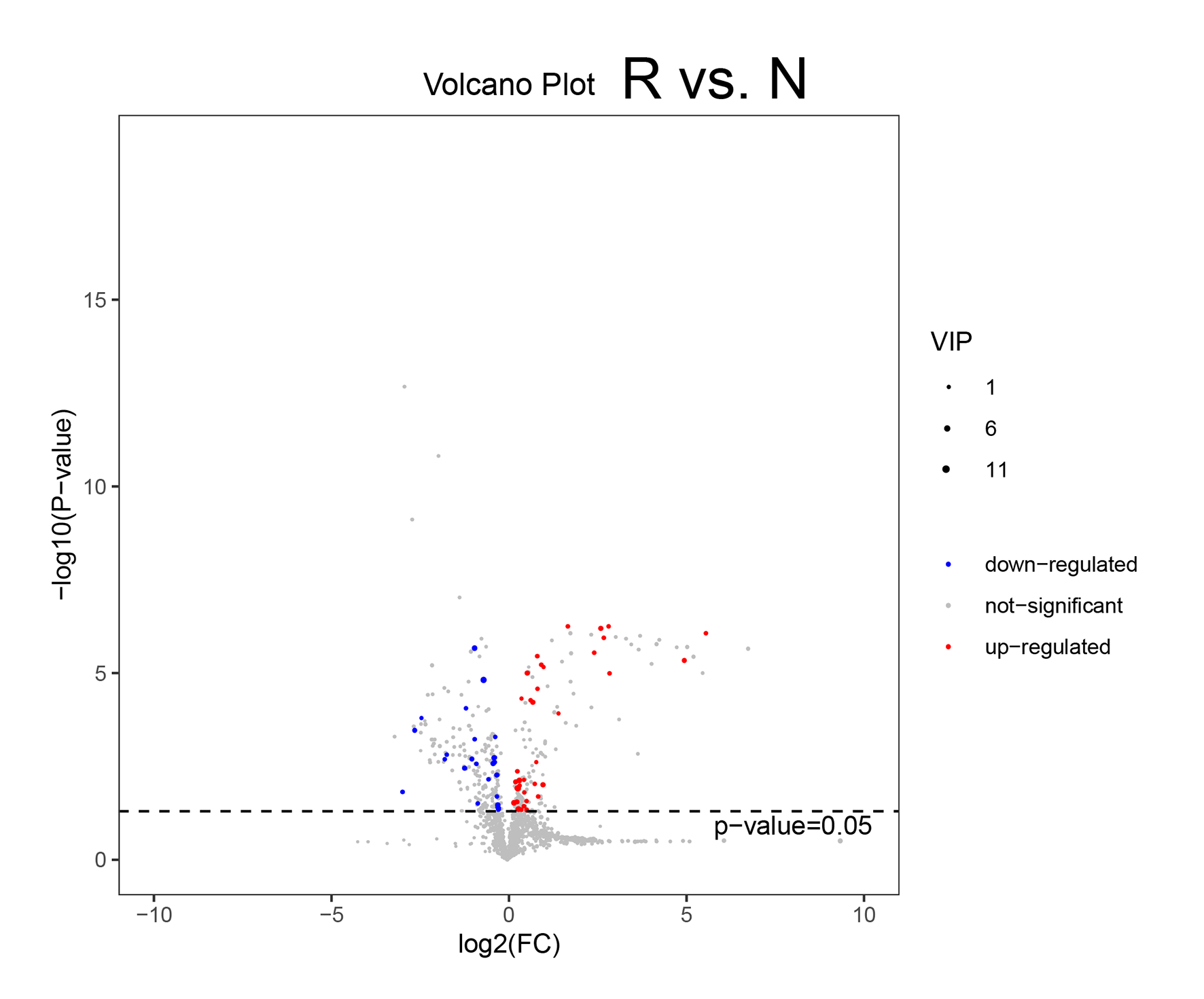

Supplement: Supplementary Figure 8 — Volcano plot demonstrating differentially expressed metabolites between the R and N groups. [file Image_8.tif]
